# Anticancer Activity of Ω-6 Fatty Acids through Increased 4-HNE in Breast Cancer Cells

**DOI:** 10.3390/cancers13246377

**Published:** 2021-12-20

**Authors:** Chhanda Bose, Ashly Hindle, Jihyun Lee, Jonathan Kopel, Sahil Tonk, Philip T. Palade, Sharad S. Singhal, Sanjay Awasthi, Sharda P. Singh

**Affiliations:** 1 Department of Internal Medicine, Division of Hematology and Oncology, Texas Tech University Health Sciences Center, Lubbock, TX 79430, USA; chhanda.bose@ttuhsc.edu (C.B.); ashly.hindle@ttuhsc.edu (A.H.); Jihyun.Lee@ttuhsc.edu (J.L.); jonathan.kopel@ttuhsc.edu (J.K.); Sahil.Tonk@ttu.edu (S.T.); 2 Department of Pharmacology and Toxicology, University of Arkansas for Medical Sciences, Little Rock, AR 72205, USA; ppalade@uams.edu; 3 Department of Medical Oncology and Therapeutic Research, City of Hope Comprehensive Cancer Center, Duarte, CA 91010, USA; ssinghal@coh.org; 4 Medical Oncology Service, Doctors Hospital, 16 Middle Rd., George Town, Grand Cayman KY1-1104, Cayman Islands, UK

**Keywords:** 4-hydroxynonenal (4-HNE), Ω-6 fatty acid, arachidonic acid (AA), doxorubicin (dox), breast cancer, Her2, p53 (TP53), Rlip76

## Abstract

**Simple Summary:**

Epidemiological evidence suggests that breast cancer risk is lowered by Ω-3 and increased by Ω-6 polyunsaturated fatty acids (PUFAs). Paradoxically, the Ω-6 PUFA metabolite 4-hydroxynonenal (4-HNE) inhibits cancer cell growth. This duality prompted us to study whether arachidonic acid (AA) would enhance doxorubicin (dox) cytotoxicity towards breast cancer cells. We found that supplementing AA or inhibiting 4-HNE metabolism potentiated doxorubicin (dox) toxicity toward Her2-dependent breast cancer but spared myocardial cells. Our results suggest that Ω-6 PUFAs could improve outcomes of dox chemotherapy in Her2-overexpressing breast cancer.

**Abstract:**

Her2-amplified breast cancers resistant to available Her2-targeted therapeutics continue to be a challenge in breast cancer therapy. Dox is the mainstay of chemotherapy of all types of breast cancer, but its usefulness is limited by cumulative cardiotoxicity. Because oxidative stress caused by dox generates the pro-apoptotic Ω-6 PUFA metabolite 4-hydroxynonenal (4-HNE), we surmised that Ω-6 PUFAs would increase the effectiveness of dox chemotherapy. Since the mercapturic acid pathway enzyme RALBP1 (also known as RLIP76 or Rlip) that limits cellular accumulation of 4-HNE also mediates dox resistance, the combination of Ω-6 PUFAs and Rlip depletion could synergistically improve the efficacy of dox. Thus, we studied the effects of the Ω-6 PUFA arachidonic acid (AA) and Rlip knockdown on the antineoplastic activity of dox towards Her2-amplified breast cancer cell lines SK-BR-3, which is sensitive to Her2 inhibitors, and AU565, which is resistant. AA increased lipid peroxidation, 4-HNE generation, apoptosis, cellular dox concentration and dox cytotoxicity in both cell lines while sparing cultured immortalized cardiomyocyte cells. The known functions of Rlip including clathrin-dependent endocytosis and dox efflux were inhibited by AA. Our results support a model in which 4-HNE generated by AA overwhelms the capacity of Rlip to defend against apoptosis caused by dox or 4-HNE. We propose that Ω-6 PUFA supplementation could improve the efficacy of dox or Rlip inhibitors for treating Her2-amplified breast cancer.

## 1. Introduction

Ω-6 and Ω-3 polyunsaturated fatty acids (PUFAs) are also called essential fatty acids, required in our diets because they cannot be synthesized de novo in higher organisms. They are precursors of bioactive eicosanoids such as leukotrienes, prostaglandins, prostacyclins and hepoxilins that are key intercellular signals for regulating immunity, cell motility and inflammation [1,2,3]. Epidemiological evidence indicates that Ω-6 fatty acids promote, and Ω-3 fatty acids prevent, cancer [4,5,6,7]. Several mechanisms have been proposed for the cancer-preventative actions of Ω-3 fatty acids, including suppression of neoplastic transformation, inhibition of cell proliferation, enhancement of apoptosis and anti-angiogenic activity. Most of these mechanisms have been directly or indirectly linked to their inhibition of the production of eicosanoids from Ω-6 fatty acids. This agrees with observations that the predominant reactive aldehyde metabolite of Ω-6 PUFA, 4-hydroxynonenal (4-HNE), is a genotoxic compound [8,9,10] that alkylates DNA bases [11,12,13] and causes DNA strand breaks to promote carcinogenesis [9,14,15,16,17].

Glutathione S-transferase (GST) isoenzymes reduce mutagenesis and delay carcinogenesis because they detoxify electrophilic mutagens such as 4-HNE. They catalyze the Michael addition reaction between glutathione (GSH) and 4-HNE to yield the thioether adduct GS-HNE [18,19,20,21,22,23]. The α-class GSTs (GSTA1-4) also prevent 4-HNE formation by catalyzing the NADPH-linked reduction of Ω-6 PUFA hydroperoxides to their corresponding alcohols, effectively eliminating the rate-limiting intermediate of lipid peroxidation [24]. 4-HNE is also metabolized through oxidation of its aldehyde group to 4-hydroxynonenoic acid (HNA) by aldehyde dehydrogenases (ALDHs) that are markers of neoplastic stem cells [25]. Alternatively, 4-HNE can be reduced to dihydroxynonenol (DHN) by aldoketoreductases (AKRs). AKR1B1 can also reduce the aldehyde group in GS-HNE to yield glutathionyl-DHN (GS-DHN), a pro-inflammatory metabolite that activates NFκB [26,27,28]. ATP-dependent efflux of GS-HNE and GS-DHN is catalyzed by glutathione-conjugated transporters such as Rlip, also known as RLIP76 (or Rlip), a protein encoded by the human RALBP1 gene (18p11.22). GS-HNE and GS-DHN are further metabolized to mercapturic acids in the kidneys and excreted in the urine [29]. Their efflux is critically important for maintaining a physiological baseline suitable for normal eicosanoid signaling because GSH conjugates are potent inhibitors of enzymes that utilize GSH to exert antioxidant effects in cells [30,31].

Small increases in baseline intracellular 4-HNE levels caused by treatment with sub-micromolar concentrations of 4-HNE increase cell proliferation [14,32,33,34], possibly through activation of EGFR signaling [32,35]. Treatment with higher concentrations inhibits proliferation by causing G2/M cell cycle arrest through inactivation of CDK1, and p53-independent activation of p21 and Chk1 [36]. Even higher concentrations cause cell death by triggering apoptosis or necrosis through GSTA4-regulated p53-dependent mechanisms involving Bax, JNK and caspase3 [37,38,39]. The thresholds for the multiphasic responses to increased intracellular 4-HNE concentrations differ between cell types, with malignant cells being generally more sensitive to the apoptotic effects than non-malignant cells. The balance between proliferation and apoptosis may rest in promoting apoptosis through FAS and ASK1 and inhibiting it through Daxx [32]. Inhibitors of 4-HNE-metabolizing enzymes including GSTA4 [14], AKR [38,40,41], ALDH [42,43,44] or Rlip [18,19,20,21,22,23,45] are selectively toxic to cancer cells.

The ability to maintain low 4-HNE levels may be a pre-requisite for the formation, survival and growth of cancer cells. Reducing the baseline 4-HNE concentration in non-malignant cells by forced overexpression of GSTA4 increases the rate of 4-HNE metabolism and triggers a dramatic switch to a malignant phenotype that is characterized by anchorage-independent growth, an increased proliferative rate, activation of numerous oncogenes and silencing of p53 [46]. We tested the logical prediction of this observation by examining the cancer susceptibility of GSTA4 knockout mice that have higher baseline levels of 4-HNE but found no reduction in their sensitivity to chemical carcinogenesis. In stark contrast, Rlip knockout mice that have increased levels of both 4-HNE and GS-HNE [47] were resistant to carcinogenesis, even by the most potent known chemical carcinogen, benzo[a]pyrene (BAP). Less than 20% of Rlip knockout mice developed lung or gastric carcinoma after a dose of BAP exposure that induced malignancy in >90% of wild-type mice [48]. Loss of one or both Rlip alleles prevents spontaneous malignancy in mice with loss of both alleles of the tumor suppressor p53 more effectively than any other previously attempted genetic or pharmacological interventions [49]. Most importantly, Rlip loss also prevented breast carcinogenesis driven by Her2 oncogenes in the Her2-overexpressing mice [50]. These findings speak to the necessity for maintaining low levels of 4-HNE for carcinogenesis and the central importance of the rate-limiting enzyme of 4-HNE metabolism, Rlip.

Interestingly, Rlip-deficient mice are resistant to carcinogenesis despite having high levels of mutagens such as lipid hydroperoxy radicals, 4-HNE and other reactive aldehydes, and consequently increased oxidative DNA damage [47,51,52]. Though observation appears to contradict the conventional paradigm of carcinogenesis due to cumulative DNA damage caused by oxidative stress, it is perhaps quite consistent with the Knudsen two-hit hypothesis concerning whether Rlip functions are necessary for the ‘second hit’ that eventually results in malignant transformation of mutated cells. Stem cell transcription factor expression, growth factor signaling, clathrin-dependent endocytosis (CDE) [48,49,50] and anti-apoptotic GSH-linked enzymes may be the key carcinogenic functions of Rlip that are required for the ‘second hit’ and are lost in Rlip-deficient mice [52]. Paradoxically, the same GSH-linked antioxidant enzymes that oppose carcinogenesis by slowing mutagenesis [53] also allow mutated cells to evade apoptosis. The loss of p53 functions accelerates carcinogenesis, and the resultant malignant cells become hopelessly addicted to the anti-apoptotic functions of these GSH-linked stress-protective enzymes. Indeed, the same mechanisms confer acquired drug and radiation resistance [54,55,56,57,58,59]. This duality underpins the cancer-selective apoptotic effects of 4-HNE and leads us to propose that Ω-6 PUFAs exert diametrically opposite effects in the settings of cancer prevention vs. treatment. Limiting the intake of Ω-6 PUFAs should aid in cancer prevention by reducing 4-HNE formation, but increasing their intake could enhance the efficacy of breast cancer treatment.

We addressed this hypothesis by comparing the effects of the Ω-6 PUFA arachidonic acid (AA) and Rlip depletion on the cytotoxicity of doxorubicin, a potent generator of 4-HNE in cancer cells [60]. Because the proliferative signals from Her2 are dependent on p53 function [61], we performed our studies in isogenic breast cancer cell lines carrying p53 mutation but differing in signaling downstream of Her2. Our results indicate that AA promotes lipid peroxidation, increases cellular 4-HNE and triggers apoptosis. AA synergistically enhanced the activity of an antisense (Rlip-LNA) that we have previously shown to regress ER+ and triple negative breast cancer xenografts [55]. AA enhanced dox toxicity synergistically in Rlip-depleted cells with relative sparing of cultured cardiomyocytes, indicating that Ω-6 PUFA supplementation may improve the effectiveness of dox chemotherapy.

## 2. Materials and Methods

### 2.1. Regulatory Compliance

The studies were performed using laboratories, equipment and safety procedures compliant with the Institutional Biosafety Committee (IBC#16013).

### 2.2. Materials

Cell lines: The human breast cancer cell lines AU565 and SK-BR-3 and rat cardiomyocyte cell line H9C2 (from rat BDIX heart myoblasts) were obtained from the American Type Culture Collection (ATCC, Manassas, VA, USA). AU565 and SK-BR-3 are both isogenic p53 mutant (p53 R175H) luminal-like Her2-expressing cell lines but differ in downstream signaling. Unlike SK-BR-3, AU565 is poorly responsive to EGF and neuregulin (NRG1β) and has higher Her3 expression, and its greater resistance to the combination of pertuzumab and lapatinib is not reversed by NRG1β. AU565 is dependent on neoplastic stem cell maintenance signaling by HGF (hepatocyte growth factor receptor) to the receptor tyrosine MET (mesenchymal epithelial transition protooncogene), while SK-BR-3 is dependent on the PIK3CA pathway [62,63,64]. Rlip depletion has been shown to inhibit PIK3CA signaling in breast cancer and epigenetically regulate MET expression [49,55,65].

Chemicals and kits: Arachidonic acid and all other chemicals used in the study were purchased from Sigma-Aldrich (St. Louis, MO, USA). RPMI 1640, DMEM, McCoy’s medium, FBS, penicillin/streptomycin, Lipofectamine 3000, Opti-MEM Medium, APO-BrdU TUNEL assay kit (cat#A35125), Dead Cell Apoptosis Kit with Annexin V Alexa Fluor 488 & PI (cat#V13245), pHrodo-red EGF Conjugate (cat#P35374), Alexa Fluor 488 complexed EGF (cat#E13345), Super-Signal West Pico PLUS Chemiluminescent Substrate (cat#34580), Pierce Clear Milk Blocking Buffer (cat#37587), 4–12% bis-tris gels, 3–8% tris acetate gels and gel running buffers were purchased from ThermoFisher Scientific (Waltham, MA, USA). CytoTox 96 Non-Radioactive Cytotoxicity Assay (cat# G1780) was purchased from Promega (Madison, WI, USA). Thiazolyl Blue reagent was purchased from Santa Cruz Biotechnology (Dallas, TX, USA), and Comet Assay Single cell gel electrophoresis Kit (cat#4251-050-K) was from R&D Systems (Minneapolis, MN, USA). 4-Hydroxy-*t*-2-nonenal (4-HNE) was assessed by OxiSelect HNE Adduct Competitive ELISA Kit (cat#STA-838, Cell Biolabs Inc., San Diego, CA). Lipid Peroxidation Assay Kit (cat#ab243377) was from Abcam (Cambridge, MA, USA), and Rlip-LNA and LNA control antisense (CAS) were purchased from Exiqon (Woburn, MA, USA) [55]. 

Antibodies: Rabbit monoclonal ALDH1L1 antibody (E7I2Q) (cat#85828S) was purchased from Cell Signaling Technology (Danvers, MA). Mouse monoclonal Aldose Reductase (C-1) (cat#sc-271007) and GAPDH (G-9) (cat#sc-365062) antibodies were both from Santacruz Biotechnology. Rabbit monoclonal antibodies for 4-HNE and GST4 were produced in house. Mouse monoclonal RalBP1 antibodies were purchased from Origene technologies (Rockville, MD, cat#TA500964) and Sigma (St. Louis, MO, USA, cat#WH0010928M2). Rabbit polyclonal beta actin antibody (cat#PA5-16914) was purchased from ThermoFisher.

### 2.3. Cell Culture

AU565 cells were maintained in RPMI 1640, SK-BR-3 cells were maintained in McCoy’s medium and H9C2 cells were maintained in DMEM medium. Growth media were supplemented with 10% FBS, 100 U/mL penicillin and 100 µg/mL streptomycin, in a 5% CO_2_ humidified incubator at 37 °C.

### 2.4. RLIP Depletion by Rlip Locked Nucleic Acid (Rlip-LNA)

Rlip was depleted by transfection using Rlip-LNA antisense. Lipofectamine complexes were prepared as follows: Rlip-LNA or scrambled control antisense (CAS) (10 µg/mL final concentration) and 4 µL of Lipofectamine 3000 reagent were separately pre-diluted in 500 μL Opti-MEM Medium and then mixed gently, incubated at room temperature for 30 min and added to plates. Cells were suspended in complete growth medium without antibiotics. After 20 min, 500 µL of cell suspension containing 1−2 × 10^6^ cells was added to each plate having an LNA oligo and Lipofectamine complex. Contents were mixed gently, and cells were incubated in a CO_2_ incubator at 37 °C. After 24 h, cells were trypsinized and centrifuged at 500× *g* for 5 min. Cell pellets were suspended in complete growth medium, counted and used for subsequent experiments as needed.

### 2.5. Cell Treatments

Cultured adherent AU565 and SK-BR-3 cells with and without Rlip depletion were trypsinized, pelleted by centrifugation at 500× *g* for 5 min at 4 °C and washed twice by suspension in complete growth medium. Live cells (lacking trypan blue) were counted using a hemocytometer. For cell viability assays, Rlip-depleted and non-depleted cell pellets were resuspended, and 1 × 10^4^ cells/well were seeded in 96-well plates before treatment with various concentrations of the Ω-6 PUFA arachidonic acid (AA) in complete growth medium.

### 2.6. Cytotoxicity Assay

After treatments and incubation at 37 °C for 48 h, cell viability was determined using the MTT assay or by lactate dehydrogenase (LDH) release assay. MTT assays were performed as described previously [66]. The cytotoxic effects of doxorubicin and AA were evaluated by measuring the percentage of LDH released by cancer cells and cardiomyocytes, which were seeded in 96-well microplates with complete growth medium containing 10% fetal bovine serum. After treatment, cell culture medium was analyzed for the percentage of lactate dehydrogenase (LDH) released using a commercially available colorimetric kit, according to the manufacturer’s instructions. Briefly, the supernatant medium (50 μL) was transferred to another 96-well plate and 50 μL of CytoTox 96 reagent was added to each well. The plate was incubated at room temperature for 30 min in the dark. An amount of 50 μL of stop solution was added to the wells, and absorption was assayed at 490 nm. Medium, volume correction and spontaneous LDH release controls were applied. Cytotoxicity was expressed as the ratio of absorption of released LDH to that of the total LDH.

### 2.7. Crystal Violet Clonogenic Assay

The effect of AA on proliferation and cytotoxicity was further tested by colony formation assay. A total of 500 cells were seeded in 6-well culture plates. Once attached, cells were treated with different concentrations of AA in complete growth medium. After 14 days, medium was removed, and the colonies were washed with PBS and then fixed using methanol and acetic acid in a 3:1 ratio. The colonies were stained with 0.5% crystal violet solution (diluted with methanol) for 5 min. The stained plates were rinsed with distilled water and allowed to air dry. Digital images of the colonies were obtained using a camera or scanning device.

### 2.8. Oxidative DNA Damage by TUNEL Assay

A terminal deoxynucleotidyl transferase dUTP nick-end labeling (TUNEL) assay was utilized to assess and validate apoptotic cell death. Flow cytometric analysis was performed using an APO-BrdU TUNEL assay kit. Rlip was depleted by Rlip-LNA, and cells were treated with 50 and 100 µM of AA. After treatments, cells were processed for staining following the manufacturer’s protocol. Cells were washed with PBS and trypsinized. Cells (1–2 × 10^6^) were suspended in 0.5 mL PBS and added to freshly prepared 1% (*w*/*v*) buffered paraformaldehyde and placed on ice. After 15 min, cells were washed twice with PBS, ice-cold 70% (*v*/*v*) ethanol was added and cells were kept on ice for 30 min. Cells were washed twice with wash buffer, resuspended in 50 µL of DNA-labeling solution and incubated for 60 min at 37 °C. Cells were rinsed two times with rinse buffer. Cell pellets were resuspended in 100 µL of antibody solution and further incubated for 30 min at room temperature in the dark. After incubation, cells were analyzed with the BD Accuri C6 Flow Cytometer (BD Biosciences, San Jose, CA, USA). The fluorescence level for discrimination between apoptotic and non-apoptotic cells was set using the control without TdT (terminal deoxynucleotidyl transferase). Cells above this fluorescence value in the TdT-positive sample were considered apoptotic. The percentages of cells undergoing apoptosis were assessed. Analysis was performed using the BD CSampler software (BD Biosciences). Viable cells were identified by gating on forward and side scatters (FSC/SSC, representing the distribution of cells in the light scatter based on size and intracellular composition, respectively). At least 10,000 cells were analyzed per staining. Data are shown as logarithmic dot plots and histograms and expressed as mean fluorescence intensity and number of counts of the positive cells obtained from the statistical analysis of the fluorescence height and the mean value of the x-axis displayed by the software [55,57]. Data were obtained from three independent experiments.

### 2.9. Comet Assay

DNA strand breaks within single cells were examined by comet assay. Assays were performed under alkaline pH conditions using the Comet Assay Single cell gel electrophoresis Kit, as per the manufacturer’s instructions. Rlip-depleted and non-depleted cells were treated with vehicle (ethanol) or with 100 µM AA for 24 h. Cells were harvested, and a cell suspension was prepared in ice-cold PBS at 1 × 10^5^ cells/mL. The cell suspension was combined with 1% low-melting point agarose at the ratio of 1:10 (*v*/*v*). An amount of 50 µL of the mixture of cells and agarose was transferred to a comet slide and allowed to set at 4 °C for 1 h. The slides were immersed in lysis solution overnight at 4 °C. Excess buffer from the slides was drained and immersed in freshly prepared alkaline DNA unwinding solution (pH > 13) for 1 h at 4 °C in the dark. Gels were electrophoresed in a horizontal electrophoresis apparatus for 40 min at 300 mA in a cold room. The samples were washed twice with distilled water for 5 min and subsequently fixed in 70% ethanol. Slides were dried at 37 °C for 15 min and stained with DAPI or ethidium bromide to visualize cellular DNA. The fluorescence images were analyzed, and DNA damage was quantified in 50 randomly selected comets per sample from two duplicate gels. The percent of DNA in the head and the tail regions of each comet was determined using the Image J OpenComet program. Three independent experiments were carried out in each case, and the means of the median values of percent tail DNA and standard deviations were calculated.

### 2.10. Apoptosis Assay by Annexin V and PI

Apoptosis in AU565 and SK-BR-3 cells was assayed by annexin V and propidium iodide (PI) co-staining using an annexin V Alexa Fluor 488 staining kit following a standard protocol. A total of 1 × 10^6^ cells were plated in a 10 cm dish, and 24 h later, cells were treated with 1, 10 and 100 µM of 4-HNE. Cells were analyzed 24h after 4-HNE treatment. Cells were harvested by addition of 0.25% trypsin and 5.3 mM EDTA for 2 min at 37 °C. Trypsin and EDTA were inactivated by addition of complete medium. Cells were collected by centrifugation at 100× *g* and resuspended in 1 mL of room-temperature annexin binding buffer (10 mM HEPES, 140 mM NaCl, 2.5 mM CaCl_2_, pH 7.4). An amount of 100 µL of the cell suspension was transferred to a flow cytometry tube containing 10 μL of Alexa Fluor 488-conjugated annexin V. The cells were incubated at room temperature for 15 min, and then 400 µL of annexin binding buffer plus 10 μL of 50 μg/mL PI was added to the cells. The stained cells were then analyzed by flow cytometry using a BD Accuri C6 Flow Cytometer (BD Biosciences, San Jose, CA, USA). The instrument was set for FL1 (annexin V) vs. FL3 (PI) bivariant analysis. Data from 10,000 cells/sample were collected, and dot plots of FL1 vs. FL3 were generated. The quadrants were set based on the population of healthy, unstained cells in untreated samples compared to cells treated with 4-HNE for 30 min. BD CSampler software (BD Biosciences) was used to calculate the percentage of the cells in the respective quadrants. A minimum of three different experiments were performed.

### 2.11. Western Blot Analysis

Total cell lysates were prepared and used for Western blots. Cell lysates (45 µg/lane) were loaded either on 4–12% bis-tris or 3–8% tris acetate gels, with 1X MES or tris acetate gel running buffer. Proteins were transferred to nitrocellulose membranes, and blocking was conducted in 1X Clear Milk Blocking Buffer with 0.1% Tween 20 for 1h at room temperature. Membranes were probed with primary antibodies against ALDH1L1, AR (AKR1B1), 4-HNE, GSTA4, Rlip, GAPDH and beta actin diluted to 1:1000 in 1× Clear Milk with 0.1% Tween 20 and incubated overnight at 4 °C using gentle shaking. Membranes were washed five times (5 min each) with tris-buffered saline-Tween 20 (TBST; 20 mM Tris·HCl (pH 7.6), 137 mM NaCl and 0.2% (*v*/*v*) Tween 20) and incubated with horseradish peroxidase-coupled anti-IgG (secondary antibody, dilution 1:2000) for 1 h, at room temperature in 1× Clear Milk with 0.1% Tween 20. For visualization of the bands, enhanced chemiluminescence (Super-Signal West Pico Chemiluminescent Substrate) was used following the manufacturer’s instructions. For the loading control, membranes were stripped with Restore Western Blot Stripping Buffer (ThermoFisher, Waltham, MA) and re-probed with anti-GAPDH or beta actin antibody (1:1000 dilution). Bands were visualized using an ImageQuant LAS4000 (GE Healthcare Life Sciences, Chicago, IL, USA).

### 2.12. Enzyme Assays

Enzymatic and GSH assays were performed on total cell lysates. GSH assay: Intracellular GSH content was determined in AU565 and SK-BR-3 cell homogenates [67]. Approximately 4–5 × 10^6^ cells were homogenized in hypotonic lysis buffer and sonicated. To 200 µL of lysate, 300 µL of precipitating solution (0.2 M glacial meta-phosphoric acid, 5 M NaCl, 5 mM EDTA) was added. The acid-precipitated proteins were pelleted by centrifugation at 4 °C for 10 min at 20,000× *g*. To determine the GSH content, 200 µL of the acid-soluble supernatants were mixed with 800 µL of 0.3 M Na_2_HPO_4_, and the initial OD was read at 412 nm. An amount of 100 µL of 0.6 mM 5, 5-dithiobis-(2-nitrobenzoic acid) (DTNB) in 1% sodium citrate was added to a final volume of 1 mL. The increase in absorption at 412 nm was monitored and used to determine the amount of GSH in the samples. Aldose reductase assay: AU565 and SK-BR-3 cell lysates were prepared in G1 buffer (20 mM potassium phosphate, 1.4 mM BME and 2 mM EDTA), and AR activity was assayed according to the method described by Reddy et al. [68]. Briefly, the assay mixture contained 50 μM potassium phosphate buffer (pH 6.2), 0.4 M lithium sulfate, 5 μM 2-mercapto ethanol, 10 μM dl-glyceraldehyde, 0.1 μM NADPH and an enzyme preparation. The assay mixture was incubated at 37 °C and initiated by the addition of NADPH at 37 °C. The change in the absorbance at 340 nm due to NADPH oxidation was measured with a SpectraMax Plus spectrophotometer (Molecular Devices). GST activity towards 4-hydroxynonenal: 4-HNE conjugating activity with glutathione was determined by methods described by Singhal et al. [69]. The assay mixture contained 100 mM potassium phosphate buffer (pH 6.5), 0.5 mM GSH and 0.1 mM 4-HNE. The blank lacked protein but included all other components, including 4-HNE (added at time 0), since the rate of non-enzymatic reaction of 4-HNE with GSH is relatively high. Measurement was started immediately after adding 4-HNE since the reaction rate is linear for a short time only [11,12,13,70]. 

### 2.13. ELISA Assay for 4-HNE–Protein Adducts

4-HNE, a biomarker of oxidative stress, was measured by the HNE–protein adducts present in cells using an ELISA kit according to the manufacturer’s instructions. In brief, 25 µg of total cell lysates was used to measure the adducts. 4-HNE–protein adducts present in the sample or standard were probed with the primary 4-HNE antibody, followed by a horseradish peroxidase (HRP)-conjugated secondary antibody. The 4-HNE–protein adduct content in the unknown samples was determined by comparing with a standard curve that was prepared from predetermined HNE-BSA standards [71].

### 2.14. Intracellular Lipid Peroxidation In Vitro Assay

Lipid peroxidation in cells was further evaluated by a ratiometric Lipid Peroxidation Sensor that changes fluorescence from red to green upon peroxidation (Lipid Peroxidation Assay (Cell-based), ABCAM) as per the manufacturer’s instructions. In brief, 5 × 10^4^ Rlip-depleted and non-depleted cells were grown in 4-chamber slides. After overnight incubation, medium was removed, and cells were treated with 100 µM AA in fresh complete growth medium for 24 h. The cells were then incubated with 1× Lipid Peroxidation Sensor for 30 min at 37 °C. The cells were washed 3 times with HBSS, and VECTASHIELD HardSet Mounting Medium, containing DAPI for nuclear staining, was used to mount the slides. Slides were imaged with a Leica fluorescence microscope. All images were taken at 400× magnification.

### 2.15. Arachidonic Acid Effect on Doxorubicin Efflux

Chemoresistance due to drug efflux is one of the major factors for failure of cancer chemotherapy. Since Rlip is the major transporter of 4-HNE, the cellular efflux and retention of doxorubicin was measured in cells treated with AA and/or Rlip inhibition by Rlip antibody. A total of 2 × 10^6^ AU565, SK-BR-3 and H9C2 cells were plated in 6-well culture plates. After overnight incubation at 37 °C in a 5% CO_2_ atmosphere, cells were exposed to 1 µM doxorubicin. After a 30 min incubation, the cells were washed with PBS followed by treatment with AA, Rlip antibody or Rlip antibody + AA for 1 h. After incubation, cells were gently washed, 5 mL of PBS was added to each well and plates were placed on an ELISA plate shaker. To test doxorubicin efflux, every 2 min for 30 min, a 50 µL aliquot of the medium from each well was taken and placed in a black 96-well flat-bottom plate. Doxorubicin fluorescence in the medium aliquots was measured using a SpectraMax iD3 Multi-Mode Microplate Reader (Molecular Devices) with 500/550 nm excitation/emission wavelengths [72]. Accumulation of dox was also examined in the cells by fluorescence microscopy. Cells (~40 × 10^3^) were plated in 4-chamber slides in complete growth medium. After 24h, medium was removed, and cells were treated with 1 µM doxorubicin in fresh medium for 30 min. After 30 min, medium was removed, and cells were washed with PBS. Cells were then treated with Rlip antibody (4 µg/mL) and/or 100 µM AA in fresh medium. After 1h, medium was removed, and cells were washed with PBS. Fresh PBS was added to each well and removed after 30 min. Doxorubicin fluorescence was imaged using a Leica fluorescence microscope at 400× magnification. Fluorescence intensity was quantified and analyzed by Image J software, version 1.46. 

### 2.16. Effect of Rlip Depletion and AA on EGF Internalization by Immunofluorescence

The effect of Rlip knockdown/depletion by Rlip-antisense locked nucleic acid (Rlip-LNA) on EGF endocytosis was studied in AU565 and SK-BR-3 cells by immunofluorescence and flow cytometry analysis. The cells were transfected with Rlip-LNA or scrambled control antisense (CAS) oligos by transfection as previously described [55,57] and treated with 50 or 150 µM AA vehicle control. After 24 h, cells were placed on ice for 20 min, then washed with cold Live Cell Imaging Solution (LCIS, ThermoFisher) containing 2 mM glucose and 1% BSA and incubated with 2 µg/mL EGF-pHrodo-red or EGF-Alexa Fluor 488 according to the manufacturer’s instructions. Cells were then incubated at 37 °C in a humidified chamber for 30 min followed by washing with LCIS. DAPI solution (0.02 µg/mL for 5 min) was used to stain nuclei. DAPI solution was removed, and citrate buffer was added to each well. Slides were analyzed using a fluorescence microscope (Olympus America, Melville, NY). Photographs taken at 400× magnification are presented. The effect of Rlip knockdown on endocytosis was further checked by flow cytometry [55,57,72]. Flow cytometry analysis: Cells were grown on 60 mm tissue culture dishes. Rlip knockdown and AA treatment were performed as described above. After 24 h, cells were trypsinized, washed with cold LCIS and counted. Cell samples (1 × 10^6^ cells) were incubated on ice for 20 min, after which the samples were centrifuged at 200× *g* for 5 min followed by washing with LCIS. After washing, cells were incubated with 2 µg/mL rhodamine-labeled EGF (EGF-pHrodo) or Alexa Fluor 488-complexed EGF (EGF-Alexa Fluor 488) in LCIS at 37 °C for 45 min, washed with cold staining buffer, incubated for 10 min at 37 °C and analyzed with a BD Accuri C6 Flow Cytometer. The excitation/emission wavelengths were 560/585 nm for EGF-pHrodo, and 488 499/520 nm for EGF-Alexa Fluor. The fluorescence level for discrimination between EGF-pHrodo- or EGF-Alexa Fluor 488-positive and negative cells was set using the unstained control. Gating for viable cells, statistical analysis and data presentation were carried out as described in Section 2.8.

### 2.17. Quantitative RT-PCR

RNA was extracted using the Qiagen (Hilden, Germany) RNeasy Mini Kit with quantification by a NanoDrop One Spectrophotometer (ThermoFisher). cDNA was synthesized using the SuperScript IV VILO Master Mix (ThermoFisher) with cDNA digestion according to the manufacturer’s protocol. Primers were either selected from the Harvard Primer Bank (https://pga.mgh.harvard.edu/primerbank/, accessed on 17 December 2021) or designed using NCBI Primer-BLAST (https://www.ncbi.nlm.nih.gov/tools/primer-blast/, accessed on 17 December 2021) and ordered from Integrated DNA Technologies (Coralville, IA, USA) or ThermoFisher. All primers were used as described previously by us [12] and verified to amplify only a single product by dissociation curves. qRT-PCR plates were analyzed using PowerUp SYBR Green Master Mix (ThermoFisher) and an Applied Biosystems QuantStudio 12K Flex Real-Time PCR System (ThermoFisher) in standard cycling mode. Wells were loaded with 300 nM of each primer and 4 ng cDNA per reaction. Ct values were determined using the default analysis settings in QuantStudio 12K Flex v1.4 software, and normalization was conducted with the ΔΔCt method.

### 2.18. Statistical Analysis

All analyses were performed using Prism 6.0 for Windows (GraphPad, San Diego, CA, USA). Results are reported as mean ± SD. The results were analyzed by a two-tailed Student *t*-test. One-way analysis of variance (ANOVA) with Tukey’s post hoc test was applied when comparing three or more groups. A *p*-value of <0.05 was considered statistically significant. Synergy between dox and AA was calculated by the combination index (CI) using CompuSyn software (CompuSyn, Inc.) and the Chou–Talalay method, using a non-constant ratio approach, where <1 indicates synergism, = 1 is an additive effect and >1 indicates antagonism.

## 3. Results

### 3.1. Effect of Rlip Depletion on AA-Mediated Cytotoxicity

**Figure 1:**
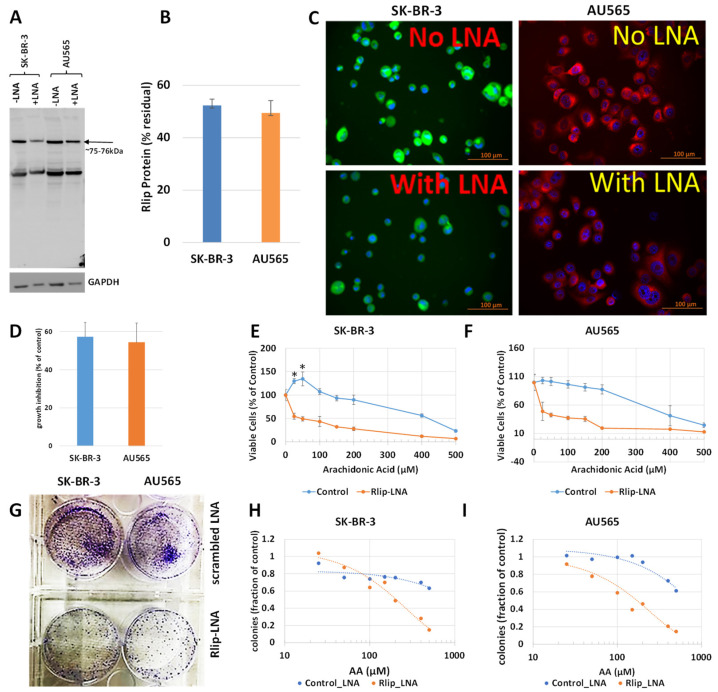
Effect of AA on p53 mutant breast cancer cell survival with and without Rlip depletion by Rlip-LNA: SK-BR-3 and AU565 cells were Rlip depleted by Rlip-LNA transfection at 24 h prior to AA treatment. (**A**) Efficiency of Rlip depletion was checked by Western blot, where GAPDH was used as a loading control for total cellular proteins. (**B**) Scanning densitometry results of Western blots showing similar levels of depletion of Rlip in SK-BR-3. (**C**) Depletion of the Rlip protein was also demonstrated by fluorescence immunocytochemistry against anti-Rlip antibodies and DAPI nuclear stain according to Materials and Methods (scale bar 100 µm). The secondary antibody used was FITC for SK-BR-3 and Texas Red for AU565 cells. (**D**) The effect of Rlip depletion by transfection of Rlip-LNA on cell proliferation was determined by MTT assay. The bar diagram shows mean ± SD of percent growth inhibition (n = 3 separate determinations) compared with control scrambled LNA. (**E**,**F**) Effect of varying concentrations of AA on cell proliferation was determined for both cell lines by MTT assay. Cells were treated with AA 24 h after transfection of either Rlip-LNA or scrambled control-LNA, and MTT assay was performed 48 h later. (**G**) Effect of Rlip-LNA on clonogenic growth, and (**H**,**I**) the effect of AA on clonogenic growth with or without Rlip-LNA. The data were analyzed by a two-tailed Student *t*-test. One-way analysis of variance (ANOVA) with Tukey’s post hoc test was applied to compare all groups. Treatment with 25 or 50 μM AA significantly increased proliferation above control in the SK-BR-3 (* *p* < 0.05) but not the AU565 cell line. (**G**) Representative images of colony formation assay as observed after crystal violet staining after Rlip-depleted and non-depleted cells were grown in culture for 14 days.

The first test of our hypothesis was to determine if AA did indeed inhibit breast cancer cell proliferation. If so, and if the effect was due to increased 4-HNE formation, Rlip depletion should enhance the inhibitory effect of AA. Because cancer suppression was observed previously even when the Rlip protein was reduced by half, we standardized the conditions for knockdown of Rlip to ~ half by Rlip-LNA. At 24 h after transfection of cells with 10 µg/mL Rlip-LNA in 0.4% Lipofectamine, cell integrity estimated by trypan blue dye exclusion was not significantly decreased. Under these conditions, the Rlip protein was reduced equally to about half of that with scrambled CAS treatment in both cell lines, as visualized in Western blots (Figure 1A) and by quantifying the Rlip band by image intensity (52.4 ± 2.4 % and 49.5 ± 4.7 % in SK-BR-3 and AU565, respectively) (Figure 1B). Immunocytochemistry using anti-Rlip antibodies qualitatively confirmed a partial depletion of the Rlip protein under these conditions (Figure 1C).

Rlip LNA was equally cytotoxic to both cell lines (48 ± 3%, n = 3) (Figure 1D). Without Rlip depletion, the three lowest concentrations of AA studied here (25–100 µM) increased the proliferation of SK-BR-3 cells by 13 ± 3.2% (*p* < 0.05) above the control. At 150 µM AA, proliferation returned to the control baseline and was decreased at all higher concentrations (Figure 1E). The effects of AA on AU565 cells resembled those on SK-BR-3, except that 25–100 µM AA did not increase proliferation above the control (Figure 1F). The IC_50_ for AA towards SK-BR-3 was numerically greater than that towards AU565 (IC_50_ 421 ± 30 and 368 ± 22 μM, respectively, 1.14×), but the difference was not statistically significant. Rlip depletion lowered the IC_50_ of AA in both cell lines by an order of magnitude: in SK-BR-3 to 35 ± 4 μM (*p* < 0.0001), and in AU565 to 23 ± 5 μM (*p* < 0.0001). Chou–Talalay analysis confirmed marked synergy between Rlip-LNA and AA (combination index 0.15). The 14-day colony forming assay demonstrated that Rlip-LNA inhibited SK-BR-3 and AU565 85.3 ± 2.7 and 91.1 ± 3.9%, respectively, more strongly than estimated by MTT assays (Figure 1G). The dose response curves for AA obtained by colony forming assays showed that AA itself reduced the colony forming potential to a degree greater than predicted by the MTT assay (*p* < 0.05) for both cell lines (Figure 1H,I). The reduction in the colony forming potential with AA and Rlip-LNA was more in agreement with the MTT results.

### 3.2. Effect of AA and Rlip Depletion on Lipid Peroxidation and 4-HNE Levels

**Figure 2:**
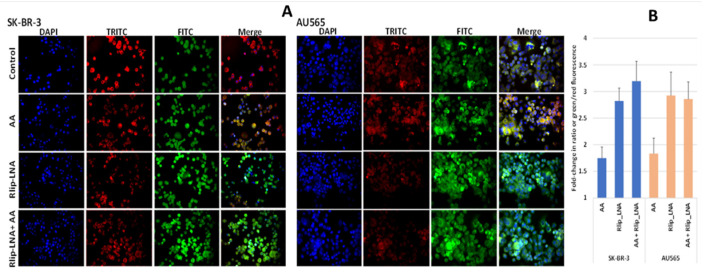
(**A**) In vitro lipid peroxidation with and without AA and Rlip-LNA treatment. Cells were first transfected with Rlip-LNA or the scrambled control (CAS), and after overnight incubation, cells were treated with 100 μM AA or vehicle control. After 24 h, lipid peroxidation was assayed using the ratiometric Lipid Peroxidation Sensor as described in Materials and Methods. Representative images are presented. All images are at 200× magnification. (**B**) Histograms represent the ratio of green to red in both cells.

Since 4-HNE is formed from oxidative degradation of Ω-6 PUFA hydroperoxides [2], we studied whether AA supplementation would cause increased lipid peroxidation (LPO) in this model system. We visualized LPO using a fluorescent ratiometric Lipid Peroxidation Sensor that changes its emission from red to green upon peroxidation by ROS in cells. Rlip depletion and AA treatment were performed as in studies above. Fluorescence micrographs demonstrated an increase in lipid peroxidation in both cell lines upon addition of 100 µM AA, evident from the increased intensity of the FITC channel and reduced intensity of the TRITC channel shown individually and as merged images from one representative study (Figure 2A). Baseline LPO was greater in AU565 compared with SK-BR-3. The ratio of green/red fluorescence estimated using Image J showed that AA alone increase LPO significantly (*p* < 0.01) in both cell lines, and Rlip LNA alone caused a larger increase (*p* < 0.01). In SK-BR-3, the addition of AA had no significant effect on LPO in either cell type (Figure 2B).

**Figure 3:**
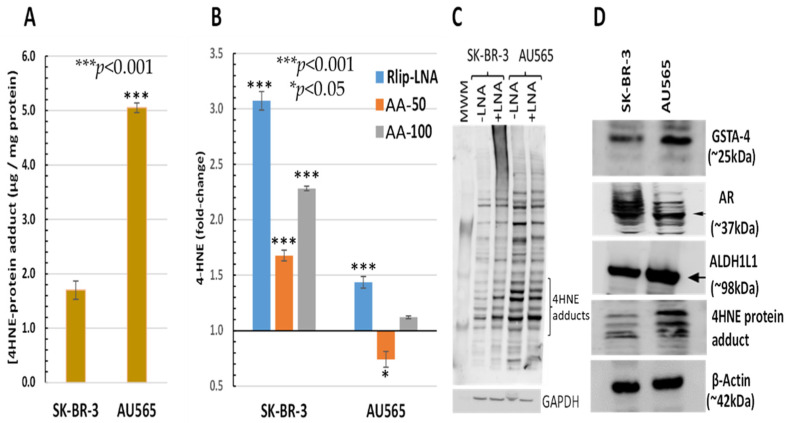
HNE–protein adducts in SK-BR-3 and AU565 cells with and without Rlip-LNA and AA. Concentration of HNE–protein adducts was determined by ELISA using protein samples from each treatment group as described in Materials and Methods. (A) Basal HNE–protein adducts were significantly higher in AU565 cells ((* *p* < 0.05, *** *p* < 0.001) than in SK-BR-3 cells. (**B**) 4-HNE was quantified by the OxiSelect ELISA assay that uses antibodies against 4-HNE–protein adducts. Cells growing in the log phase were treated with either Rlip-LNA or corresponding scrambled control LNA (10 μg/mL in 0.4% Lipofectamine) for 24 h prior to assay. Rlip depletion significantly (*p* < 0.001) increased 4-HNE in both cell lines. 4-HNE was increased significantly over control after treatment with either 50 or 100 μM AA in SK-BR-3 (*p* < 0.001, n = 3 independent measurements with 8 replicates each). AA actually reduced 4-HNE in AU565 (*p* < 0.05), but 100 μM caused a small increase (*p* < 0.05). (**C**) The effect of Rlip depletion by Rlip-LNA was visualized by Western blots against 4-HNE–protein adduct antibodies on total cell lysates, returning similar qualitative results compared with the ELISA assay. (**D**) Western blots are shown to visualize differences in antigenic levels of GSTA4, AR and ALDH1 (using ALDH1L1 antibodies cross-reactive with ALDH1). All Western blots shown are representative of results from three separate experiments. Uncropped Western blots figures are shown in Figure S2.

The AU565 cell line, which has a lower IC_50_ for AA and lacks the proliferative effect at low concentrations, had a 2.97 ± 0.51-fold higher 4-HNE level than SK-BR-3 (Figure 3A). Because of the relatively sharp cutoff between the proliferative and inhibitory effects between 50 and 100 μM AA under the present conditions, we compared the effects of treatment with 50 and 100 μM AA. The 4-HNE level increased by 68 and 128% (*p* < 0.001) upon treatment of SK-BR-3 with 50 and 100 μM AA, respectively. In contrast, the 4-HNE level unexpectedly decreased by 26% (*p* < 0.05) at the lower AA concentration but increased by 12% (*p* < 0.05) at the higher AA concentration in AU565. In both cell lines, Rlip depletion caused a significant increase in 4-HNE (40 vs. 310% in AU565 vs. SK-BR-3, respectively, *p* < 0.001) (Figure 3B). The rise in 4-HNE–protein adducts was confirmed by Western blots against antibodies specific for 4-HNE–protein adducts. Consistent with the ELISA assay, the 4-HNE adducts were greater at baseline (-LNA) in AU565 than SK-BR-3 and were less affected by Rlip depletion (+ LNA) in the latter (Figure 3C). Antigenic levels of GSTA4-4 and ALDH1L1 were greater in AU565 than SK-BR-3, but AR was higher in SK-BR-3 (Figure 3D). GST activity in conjugating 4-HNE with GSH and AR activity in reducing 4-HNE to DHN was changed in parallel with the antigen level, the former greater in SK-BR-3 and the latter in AU565 (Table 1).

### 3.3. Apoptosis by Rlip Depletion: AA or 4-HNE

The AA concentrations that increased lipid peroxidation and 4-HNE synergistically enhanced growth inhibition by Rlip-LNA. As shown by the HNE–protein adducts in Figure 3, AU565 had higher baseline 4-HNE which increased minimally in response to 100 μM AA. 4-HNE increased by 2-fold in SK-BR-3 which had lower baseline 4-HNE; unlike AU565, 100 μM AA increased the proliferation of SK-BR-3. Indeed, 100 μM AA reduced the clonogenicity of AU565 more than that of SK-BR-3. These findings suggest that the various effects exerted by 4-HNE have somewhat differing thresholds specific for each cell line.

**Figure 4:**
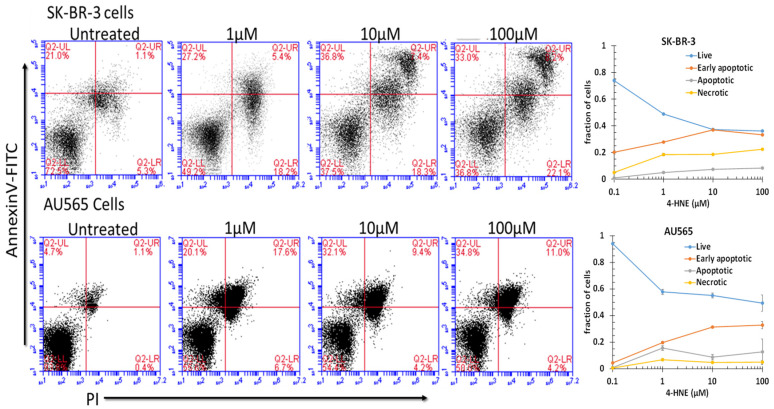
Role of 4-HNE in cancer cell death. To check the effect of 4-HNE on SK-BR-3 and AU565 cell survival, apoptotic cell death and necrotic cell death were measured using annexin and PI staining by flow cytometry. Cells were treated with different concentrations of 4-HNE for 24 h and processed as described in Materials and Methods. Viable cells were identified by gating on forward and side scatters. The logarithmic dot plots show the annexin- and PI-positive cells cluster in distinct groups after 4-HNE exposure. Quantitative analysis of the percent of cells in each gate after treatment is presented as the mean ± SD of three independent experiments.

Because the free-4-HNE concentrations in cells have been estimated to range from 0.1 to 0.3 μM [73], we assumed that AA treatment at a concentration near the threshold of toxicity could increase intracellular levels to near 1 μM under the present conditions. We found that treatment with 1 μM 4-HNE resulted in a detectable and significant (*p* < 0.001) increase in pre-apoptosis in both cell lines (Figure 4). Significant increases in late apoptosis and necrosis were also apparent in SK-BR-3 cells (*p* < 0.05), but AU565 cells were resistant to necrosis. There was a dose-dependent increase in necrosis, with the largest increase between 10 and 100 μM (*p* < 0.05). The differences in necrosis and early or late apoptosis between 10 and 100 μM were surprisingly small and not significant under the present conditions at the 24 h time point after treatment. These findings indicate that both cell lines had a low threshold for initiation of apoptosis, but necrosis depended on a larger magnitude of increase that may have been prevented by the nearly 4-fold higher GSTA4 activity and higher Rlip protein in AU565. Though AR was somewhat higher in SK-BR-3, metabolism to DHN is perhaps not as protective. The HNE concentration was increased to 10 or 100 μM, suggesting a threshold effect. The SK-BR-3 cell line was also distinguished from AU565 by the appearance of a distinct double-positive population. The known complex hypodiploidy of SK-BR-3 cells suggests that this may be due to karyotypically distinct subpopulations with differential susceptibility to apoptosis [74] or perhaps the formation of cell doublets.

**Figure 5:**
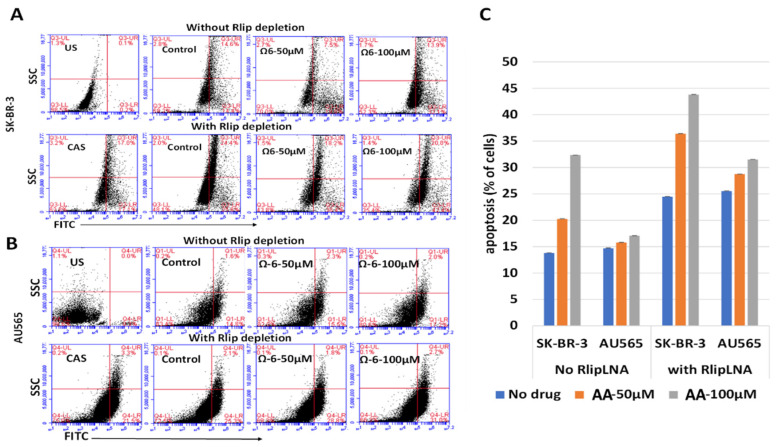
(**A**–**C**) Effect of AA and Rlip-LNA on apoptosis by terminal deoxynucleotidyl transferase dUTP-mediated nick-end labeling (TUNEL) assay. (**A**,**B**) TUNEL analysis after AA (treated for 24 h) with and without Rlip depletion. Cells were transfected with Rlip-LNA or control antisense (CAS) as described in Materials and Methods. After Rlip depletion and/or Ω6 (AA) treatment, the apoptotic intensity was determined by flow cytometric TUNEL assay. Logarithmic dot plots show the percentage of TUNEL-positive cells in different groups (US—unstained) as measured by flow cytometry. Viable cells were identified by gating on forward and side scatters (FSC/SSC, representing the distribution of cells in the light scatter based on size and intracellular composition, respectively). (**C**) Quantitative analysis of the counts of TUNEL-positive cells of each treatment is presented as the mean of three independent experiments. The fluorescence level for discrimination between apoptotic and non-apoptotic cells was set using the control without TdT (terminal deoxynucleotidyl transferase). Cells above this fluorescence value in the TdT-positive sample were considered apoptotic. Analysis was performed using the BD CSampler software (BD Biosciences). At least 10,000 cells were analyzed per staining. Data were obtained from three independent experiments.

Because the annexin V/propidium iodide assay is not designed to detect the execution phase of apoptosis, we used a flow cytometric TUNEL assay for examining the cause of cytotoxicity of AA. We confirmed that AA caused apoptosis at doses of AA that appeared nontoxic in the MTT assay, that SK-BR-3 was more sensitive to the effects of AU565 and that depletion of Rlip significantly enhanced the apoptotic effect of AA in both cell lines (Figure 5). The TUNEL results were consistent with the results of the clonogenic assay and annexin but not entirely with the MTT results. The underestimation of AA cytotoxicity by the MTT assay is likely methodological because it indirectly estimates cell viability based on mitochondrial respiration.

**Figure 6:**
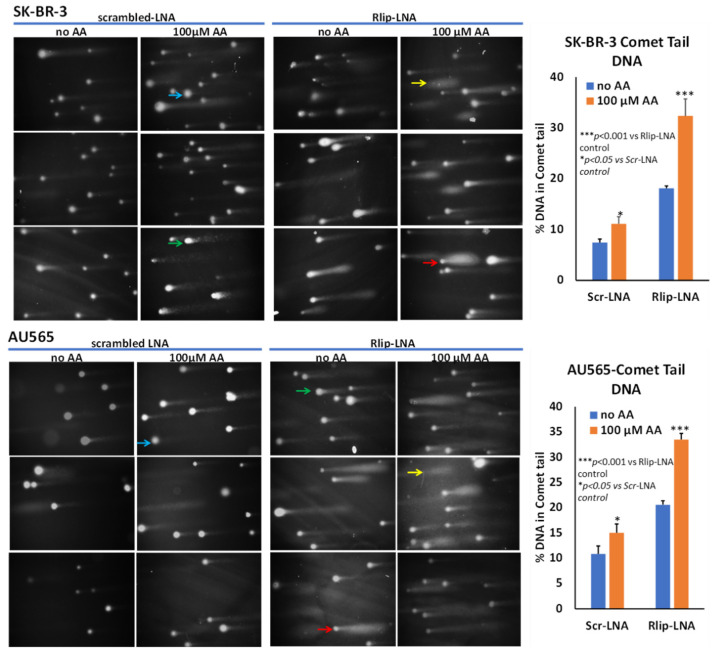
Effect of AA after Rlip-LNA on DNA fragmentation in SK-BR-3 and AU565 cells by comet assay. Representative images at 200× magnification of alkaline comet assays from control, and 100 μM AA-treated SK-BR-3 and AU565 cells with or without Rlip knockdown. Blue arrows point to cells with enlarged nuclei with minimal damage and a faint tail; green arrows point to typical comet appearance; red arrows point to ‘hedgehog’ comets; and yellow shows only a cloud of fragmented DNA. Image J was used to quantify the intensity of the DNA tail defined to the right of the nucleus regardless of shape. A two-tailed unpaired Student *t*-test was used to calculate the significance.

In addition to triggering apoptosis, lipid peroxidation also exerts genotoxicity through mutations as well as single- and double-strand DNA cleavage which can be reversible if normally functioning p53 protein can stop cell cycling to allow DNA repair. We examined DNA damage by an alkaline comet assay to estimate DNA fragmentation that appears as a ‘comet tail’ when cells embedded in agarose are electrophoresed and stained. Our results confirmed the appearance of apoptosis, as indicated by comets with varying tail lengths of DNA. The shape of the comet tail has previously been proposed to distinguish apoptosis from potentially reversible genotoxic injury, but whether this distinction is possible is debated [75]. Regardless, the shape of the comets was consistent with varying degrees of DNA damage (Figure 6). AA treatment alone caused the appearance of comets to a lesser degree than Rlip-LNA, and the combination resulted in a marked increase in comets (*p* < 0.001). Interestingly, this assay indicated a very similar susceptibility of both cells, aligning with the results of the MTT assay. Together, the three assays showed that AA caused apoptosis that was enhanced by Rlip depletion.

### 3.4. Effect of AA and Rlip Depletion on Doxorubicin Cytotoxicity in Cancer Cells and Cardiomyocytes

Since adding AA to the medium increased lipid peroxidation and 4-HNE levels and potentiated apoptosis caused by Rlip depletion, we surmised that the combination should synergistically enhance cell killing by dox, a drug known to promote lipid peroxidation. For potential clinical applicability, the combination would have to be selective for cancer cells, sparing cardiomyocytes. Thus, we compared the cytotoxic effects of the combination of AA, Rlip-LNA and dox between breast cancer cell lines and the immortalized H9C2 cardiomyocyte cell line.

**Figure 7:**
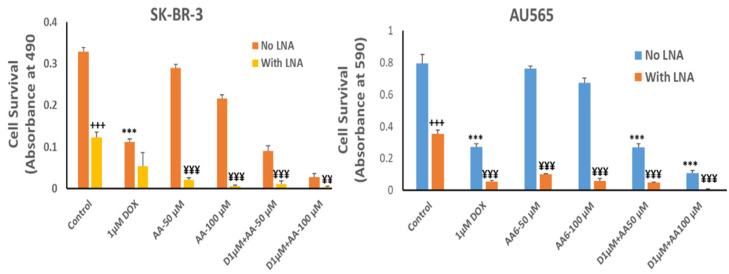
Effects of arachidonic acid and Rlip knockdown on doxorubicin toxicity in breast cancer cells. SK-BR-3 and AU565 cells were Rlip depleted by Rlip-LNA as described above and treated with 1 µM dox (labeled as D in the figures) and 50 or 100 µM AA, alone or in combination, as described in Materials and Methods. After 24 h, cells were analyzed for survival by MTT assay. The data were analyzed by a two-tailed Student *t*-test. One-way analysis of variance (ANOVA) with Tukey’s post hoc test was applied to compare all groups. The expressed values are means ± SD (n = 3 independent experiments with eight replicates). ^+++^ *p* < 0.001, no LNA vs. LNA control (CAS); *** *p* < 0.001, comparison between no LNA control and AA treatments; ^¥¥^ *p* < 0.01, ^¥¥¥^ *p* < 0.001, comparison between LNA control and AA treatments, as analyzed by a two-tailed Student *t*-test.

The growth inhibitory effect of depleting Rlip to ~half with Rlip-LNA was equivalent to 1 µM dox (Figure 7, comparing second and third bars from each cell type). Rlip depletion increased dox toxicity, but the combination (fourth bar) was not synergistic. In the absence of Rlip depletion, the effects of AA and dox were additive (compare bars 3, 5 and 9 and bars 3, 7 and 11), without synergy. In Rlip-depleted cells, synergy was observed between AA and dox (compare bars 4, 6 and 10 and bars 4, 8 and 12), with a combination index of <1 (0.59 and 0.02 in SK-BR-3 and AU565 cell lines, respectively) according to the Chou–Talalay analysis.

**Figure 8:**
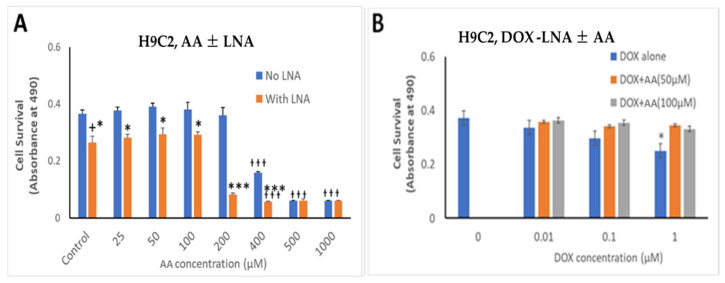
Effects of AA and Rlip knockdown on doxorubicin toxicity in the immortalized H9C2 cardiomyocyte cell line. H9C2 cells were transfected with Rlip-LNA or scrambled control LNA as described above. Results of MTT assay are shown in cells (**A**) treated with either Rlip-LNA or scrambled control and AA at 25–1000 µM or (**B**) with 1 µM dox with 50 or 100 µM AA. After 24 h, cells were analyzed for survival by MTT assay. The expressed values are means ± SD (n = 3 independent experiments with eight replicates). + *p* < 0.05, no LNA vs. LNA control; * *p* < 0.05, *** *p* < 0.001, comparison of AA treatments between no LNA and LNA; ^ƚƚƚ^ *p* < 0.001, comparison between AA treatments with their corresponding controls (0), as analyzed by a two-tailed Student *t*-test.

The effects of Rlip depletion combined with AA were also studied in cardiomyocytes by the MTT assay. Rlip-LNA depleted the Rlip protein nearly completely in the H9C2 cells, unlike in the cancer cells (data not presented) under the same conditions. The concentration range of dox was chosen between 10 and 1000 nM because the standard intravenous dose of doxorubicin of 50–60 mg/m^2^ used to treat breast cancer provides a peak serum concentration of 1000–1200 nM and decreases to 50 -100 nM within 24 h [76]. The IC50 for dox in most breast cancers ranges from 100 nM. We observed that Rlip depletion significantly reduced cardiomyocyte growth by 23%, comparing bars 1 and 2 in Figure 8A (*p* < 0.05), but addition of AA up to 100 µM did not have any additional effect (Figure 8, bars 2, 4, 6 and 8). Above 200 µM, AA inhibited their growth substantially (orange bars at 400, 500 and 1000 µM), even in the absence of Rlip depletion (blue bars at 400, 500 and 1000 µM), and Rlip depletion exacerbated this toxicity at 200 and 400 µM (*p* < 0.001). However, in the absence of Rlip depletion, concentrations of AA up to 200 µM did not inhibit the growth of this immortalized cardiomyocyte cell line, indicating a significant selectivity of the combination of AA and dox for cancer cells.

**Figure 9:**
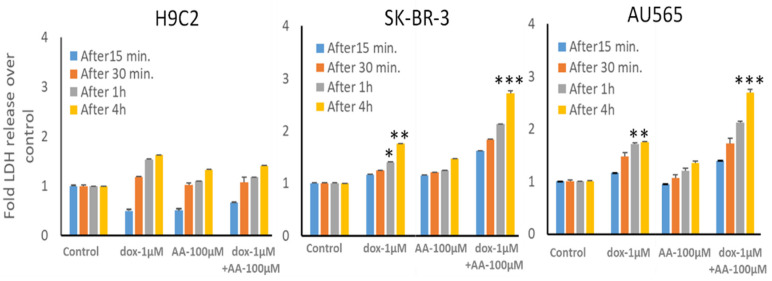
LDH release from H9C2 cardiomyocyte and SK-BR-3 and AU565 breast cancer cell lines by the combination of arachidonic acid and doxorubicin. LDH release by cells after treatment with 1 µM dox and/or 100 µM AA was performed as described in the Materials and Methods. Following treatments, media were collected from these cultures at various time points and analyzed for LDH release. The data presented show the time dependence of LDH release in all cell types compared to untreated control cells. The expressed values are means ± SD (n = 3 independent experiments with eight replicates; * *p* < 0.05, ** *p* < 0.01, *** *p* < 0.001).

The cardiomyocyte cell line used here is immortalized, unlike cardiomyocytes that do not proliferate in vivo; thus, these results cannot be easily extrapolated to predict the in vivo cardiotoxicity of the combination. Accordingly, we performed LDH release assays to assess whether cellular integrity is acutely compromised by AA across a clinically relevant range of dox concentrations from its peak until it drops below therapeutic levels. Because even 10 nM dox can significantly reduce clonal growth in breast and other cancers, we measured LDH release by the effect of the addition of 0.01 to 1 µM dox. Dox alone caused a time- and concentration-dependent release of LDH (Figure 9, H9C2 panel, bars 5–8), confirming the known acute toxic effects of dox on cardiac myocytes attributed to lipid peroxidation due to redox cycling of the quinone ring of dox [76,77]. Addition of 100 µM AA also caused LDH release, but less than dox. We observed a significantly enhanced rate of LDH release from both breast cancer cell lines caused by the addition of 100 µM AA with 1 μM dox (*p* < 0.001 at the 4 h time point). These results indicate that concentrations of AA that enhance dox effects in breast cancer cells could spare cardiac cells.

### 3.5. Effect of AA and Rlip Transport Inhibition on Accumulation and Efflux of Doxorubicin

Rlip has been shown to catalyze the ATP hydrolysis-dependent, anti-gradient, transmembrane efflux of dox [78,79,80] and is a recognized determinant of dox pharmacology [81]. Similarly, Rlip catalyzes the efflux of the glutathione conjugate of 4-HNE (GS-HNE), a mercapturic acid precursor. Indeed, each compound competitively inhibits the efflux of the other [82,83,84]. If the enhancement of the anticancer activity of dox is mediated through HNE formation and competitive inhibition of dox efflux by GS-HNE, there should be a demonstrable reduction in dox efflux and increased cellular retention of dox upon concomitant AA treatment. We exploited the fluorescence of dox to measure cellular retention and efflux following treatment with AA without or with inhibition of dox transport by a monoclonal anti-Rlip antibody (RlipAb) in SK-BR-3 and AU565 cells and H9C2 cardiomyocytes. The dox efflux rate was determined by exposing cells to 1 µM dox alone for 30 min, followed by a change to fresh growth medium containing RlipAb or 150 µM AA for 1 h. Effluxed dox was then quantified by fluorescence in growth medium collected over 30 min.

**Figure 10:**
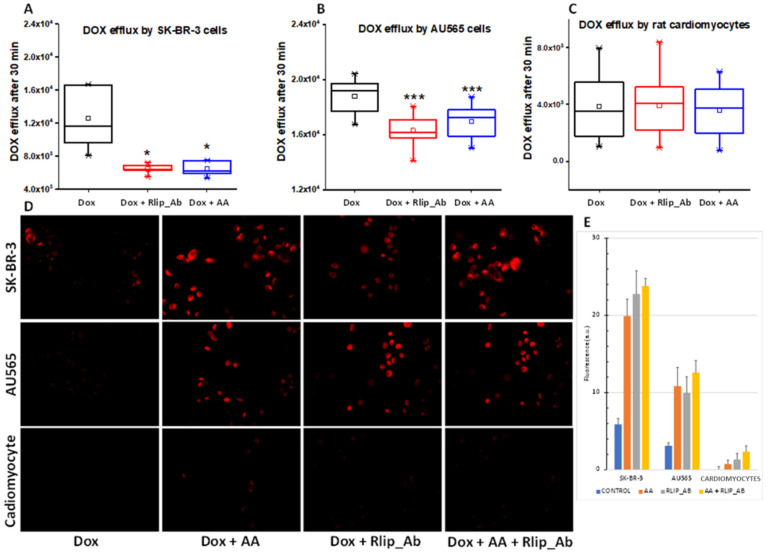
(**A**–**E**) Doxorubicin efflux after AA in SK-BR-3 and AU565 cells and H9C2 cardiomyocytes. The assay was performed as described in Materials and Methods. (**A**) Box and whisker plots show dox effluxed into growth medium. Following a 30 min dox exposure and a 1 h exposure to Rlip antibody or 150 µM AA, efflux was measured by timed sampling of the medium over 30 min. Data presented here are representative of three independent experiments. * *p* < 0.05, *** *p* < 0.001, as compared to dox alone. Box: 25–75 percentiles; whiskers: 1.5 IQR; horizontal inner line: median; small square: mean. The significance of differences among groups was evaluated using one-way analysis of variance (ANOVA) with a post hoc Tukey–Kramer test. All images are at 400× magnification. Histograms show quantitated fluorescence intensity as measured by Image J.

AA treatment significantly decreased the efflux of dox in both cancer cell lines (Figure 10A,B, blue vs. black, *p* < 0.05). It did not affect dox efflux from cardiomyocytes (Figure 10C), although these cells showed a very low basal level of dox efflux relative to the breast cancer cells. RlipAb strongly inhibited efflux of dox from the breast cancer cells (Figure 10A,B, *p* < 0.001) but had no significant effect on dox efflux from the cardiomyocytes (Figure 10A–C, red vs. black). These results were corroborated by the increased retention of dox in these cells, as shown by quantifying dox by fluorescent imaging of the breast cancer cells. In this assay, a small but significant increase in dox retention was observed in cardiomyocytes (*p* < 0.05) by the combination of all three treatments (Figure 10D,E).

### 3.6. Effect of AA and Rlip Transport Inhibition on Endocytosis of EGF

The ATPase activity of Rlip is also a rate determinant of CDE that functions in the internalization of the EGF–EGFR complexes from the plasma membrane into intracellular clathrin-coated vesicles, upon which the receptor tyrosine kinase signaling complex is assembled. CDE-mediated internalization of EGF is reduced by ~80% in Rlip null mice [49,55]. Signaling through all major pathways that promote cancer growth downstream of EGF is also impaired similarly, contributing to the anticancer effects of Rlip inhibition [49,55,57,85]. Because this endocytic function is coupled to the efflux of GS-HNE, we investigated the effect of AA and Rlip-LNA on the endocytosis of fluorescence-labeled EGF.

**Figure 11:**
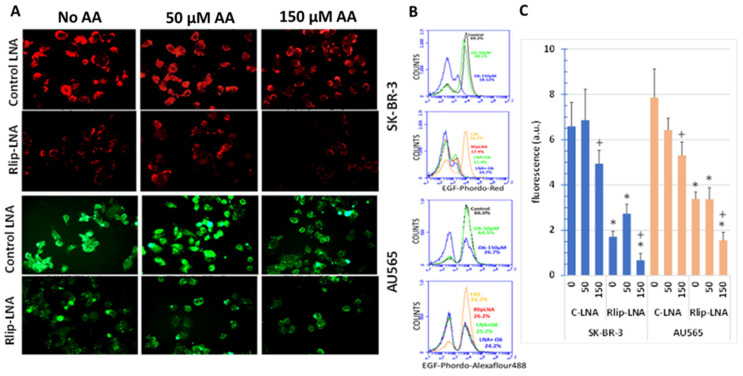
Effect of AA treatment on EGF binding and internalization after Rlip depletion: cells were transfected with Rlip-LNA or control LNA antisense (CAS) and treated with AA as described in Materials and Methods. (**A**) Representative images from three separate experiments show EGF-pHrodo-red (SK-BR-3 cells) and EGF-pHrodo-Alexa Fluor 488-green (AU565 cells) for EGF internalization after AA treatments, with and without Rlip depletion. All images are at 400× maginification. (**B**) Cells were treated identically to those in panel (**A**) and analyzed by flow cytometry. (**C**) Quantitative analysis of fluorescence intensity is the mean ± SD of three independent experiments (+ *p* < 0.05 vs. no C-LNA, * *p* < 0.001 vs. respective C-LNA concentrations).

Rlip-LNA significantly reduced EGF-pHrodo, as visualized by fluorescence imaging (Figure 11A). Diffuse cytoplasmic staining was replaced by punctate staining accumulated at plasma membranes and adjacent sub-membrane vesicles. When more clearly visualized by EGF-Alexa Fluor 488 endocytic imaging of AU565 cells, we found that treatment with AA alone changed the overall appearance of the AU565 cells to resemble cells with only Rlip-LNA treatment. These results provided strong visual evidence that AA supplementation at 150 μM transforms the EGF endocytic phenotype to resemble that of Rlip-depleted cells (compare top right to second row middle for each cell type). Combined treatment caused no additional effect over either treatment alone. Characteristically, Rlip depletion resulted in polarization and clustering of EGF on the membrane, and a similar effect was evident at 150 μM, but not at 50 μM AA. Treatment with 50 μM AA increased the number of binucleate cells, in agreement with the increased proliferation at this concentration from the MTT results.

Endocytosis quantified by flow cytometry of EGF-pHrodo-stained SK-BR-3 cells confirmed that 50 μM AA had relatively little effect on the population of cells with the highest intensity of staining, while 150 μM AA essentially abrogated this population (Figure 11B). Total fluorescence was also not significantly affected by 50 μM AA but was reduced significantly by 150 μM AA (Figure 11C). Endocytosis quantified by flow cytometry of EGF-Alexa Fluor 488-stained AU565 cells yielded similar results, with minimal effect at 50 μM, but abrogation of the highly fluorescent cell population at 150 μM AA.

### 3.7. Effect of AA on Expression of Antioxidant Genes

We studied the effect of treatment with 100 µM AA on the expression of selected antioxidant genes by qRT-PCR. AA caused the differential expression of seven of these genes in SK-BR-3 and three in AU-565. AKR1C3, GCLM and GCLC were significantly upregulated by AA in both cell lines, with AKR1C3 being affected most. SOD1 and GPX1 were upregulated in SK-BR-3, but not in AU565. SOD2 and AKR7A2 were downregulated in SK-BR-3, but not in AU565. Expression of the transcription factor NFE2L2 (NRF2), which broadly controls a large set of genes altered in response to oxidative stress, was unchanged in both cell lines. GSTA4, known to be transcriptionally regulated by NRF2, was also unaffected in both cell lines (Table 2). Based on these findings, reductive metabolism of AA and GSH synthesis would increase in both cell lines.

## 4. Discussion

We have demonstrated, for the first time, that AA supplementation inhibits the proliferation of Her2-overexpressing p53 mutant breast cancer cell lines at subtoxic concentrations that enhance dox cytotoxicity, with relative sparing of cardiomyocytes. AA supplementation increases lipid peroxidation, 4-HNE formation and dox accumulation and inhibits endocytosis of EGF. The predominant mechanism of cell death by either AA, 4-HNE or Rlip-LNA is through apoptosis, and the combinations are primarily synergistic.

The effects of AA on LPO, 4-HNE levels, apoptosis and efflux as well as cytotoxicity were similar to those of Rlip-LNA in both cell lines despite their differences in resistance to Her2-targeted therapeutics or downstream signaling pathways. AU565 is known to have higher Her3 expression and greater resistance to trastuzumab, pertuzumab and lapatinib in comparison with SK-BR-3. The resistance of AU565 to these therapies that target Her2 signaling appears to be independent of tumor microenvironment signaling by neuregulin (NRG1β). Activation of Met by HGF is more important in AU565, while the SK-BR-3 pathway depends more on PIK3CA signaling [62,63]. Rlip depletion has been shown to inhibit PIK3CA in other cancers [55,86,87], and both Rlip and PIK3CA are known to regulate CDE [55,88,89,90]. Indeed, signaling initiated by growth factors including the EGF family (including Her3) and HGF is known to be broadly regulated by endocytosis of the receptor/ligand complex by CDE [91,92], and thus also by Rlip. The AP2 clathrin adapter protein of the clathrin-coated vesicles binds Rlip [93], and the ATPase and GS–E (glutathione–electrophile conjugate) transport activity of Rlip is coupled to CDE. Rlip null mice have severely impaired CDE, and depleting Rlip by an antisense in cultured cells also inhibits CDE. Using point mutants of Rlip, we have shown that the rate of internalization of EGF in cultured cancer cells is decreased in proportion to the transport activity of Rlip [90]. The present studies of EGF endocytosis by two different fluorescent tags confirmed the known dependence of EGF endocytosis on Rlip. Both cell lines were sensitive to endocytosis inhibition by Rlip-LNA. The greater reduction in SK-BR-3 cells was likely a combined effect of CDE inhibition and apoptosis, which occurs earlier and to a greater extent in SK-BR-3 compared with AU565 cells.

Interestingly, 50 μM AA alone did not reduce EGF uptake, but 150 μM AA was significantly inhibitory. Indeed, fluorescence internalization by SK-BR-3 cells was numerically greater than the control at 50 μM AA, though not statistically significant (*p* < 0.1). These findings suggest a threshold concentration above which the effects of 4-HNE switch from proliferation to growth inhibition or apoptosis. The switch between proliferation and apoptosis could be the balance between the rate of GS-HNE formation and rate of its disposition governed by the enzyme kinetics of Rlip. If the intracellular concentration of GS-HNE approximates its K_m_ for transport by Rlip, small increases would increase the efflux rate, but the GS-HNE concentration above the requirement for reaching V_max_ would exceed the efflux capacity. Because GS-HNE (glutathione–HNE conjugate) is formed through a Michael addition reaction that is reversible in the presence of GST enzymes [94], accumulation of GS-HNE would translate to accumulation of intracellular 4-HNE. Since the off-rate of GS–E is relatively low, it is subject to product inhibition [95]; thus, accumulation of GS-HNE would inhibit GST activity. Additionally, inhibition of glutathione reductase by GS–E [96] would lower GSH and impair the reduction of hydroperoxides by GPx (glutathione peroxidase). The combined effect would be an increase in electrophilic toxins such as 4-HNE as well as oxidant and free radical toxins that initiate or originate from LPO (lipid peroxides).

AA supplementation without or with Rlip depletion was nearly equally as cytotoxic to both cell types that clearly differed in their baseline 4-HNE level and in their sensitivity to LPO. The baseline LPO was lower, and the increment in LPO caused by AA was of a lesser magnitude in SK-BR-3 than that in AU565, but the magnitude of increase in 4-HNE was greater in SK-BR-3. The greater baseline LPO in AU565 implies less effective quenching of oxygen or lipid hydroperoxy radicals by the combined effects of SOD, CAT and GPx in AU565. Higher GSTA4 and lower AR activity would divert 4-HNE away from reductive metabolism to DHN (dihydroxynonenol) by AR and towards GSTA4-catalyzed formation of GS-HNE, thus causing a greater dependence on Rlip to control 4-HNE levels. Higher ALDH in AU565 would metabolize 4-HNE to 4-HNA, blunting the rise in cellular 4-HNE caused by treatment with AA. However, higher ALDH in AU565 may potentiate its stem cell-like behavior as in other cancers [97].

The importance of 4-HNE-metabolizing enzymes is supported by the antineoplastic activities of inhibitors of AR, GSTA4 and ALDH as well as Rlip [14,41,42,44,98,99,100]. Indeed, the differences in their expression could potentially be exploited as biomarkers for selecting treatments for Her2-amplified breast cancer. For instance, AR inhibitors, which can be cardioprotective [99,101], would be predicted to be more effective in Her2-amplified breast cancer responsive to trastuzumab/pertuzumab/lapatinib, while inhibitors of Rlip should be more effective in AU565 that should generate more GS-HNE. Additionally, ALDH inhibitors could be used in cells resistant to Her2 signaling inhibitors. The differences between AU565 and SK-BR-3 in AA-induced transcriptional regulation of other antioxidant enzymes, such as SOD and GPX that determine the intensity of lipid peroxidation, could also serve as predictive biomarkers for selecting therapy for Her2-amplified breast cancer. The differences in LPO between SK-BR-3 and AU565 can certainly be due to differential formation of other toxic eicosanoids, but the GSH-linked antioxidant enzymes and Rlip serve broadly as defenses against these as well.

The cytotoxicity of AA in Her2-positive cell lines at concentrations that spare cardiomyocytes suggests that dietary AA could improve outcomes for treatment of Her2-positive breast cancer. The prevention of Her2-driven breast carcinogenesis by Rlip deficiency [50] and similarities in the effects of AA and Rlip-LNA lead us to a provocative possibility that a relative deficiency of Ω-6 essential fatty acids predisposes to Her2-positive breast cancer and that their supplementation could be preventative. One in five breast cancers overexpress Her2, but risk factors remain obscure [102]. Polyunsaturated fatty acid intake appears to modulate spontaneous carcinogenesis in Her2-amplified mice [103]. Perhaps the rising incidence of Her2-positive breast cancer [104] is related to reduced consumption of Ω-6 fatty acids, but whether they alone promote inflammatory or cardiovascular disease remains controversial [105].

Low concentrations of AA increased the activity of dox towards breast cancer cells, with significantly less cytotoxicity towards cardiomyocytes in proliferation or LDH release assays. Rlip is known to catalyze the efflux of dox from cancer cells, and mutually competitive inhibition has been shown between dox and GS–E [80,106], but other dox transporters may be more important in cardiomyocytes. Specific inhibitors of other dox transporters (ABCB1, ABCC1) are known to cause cardiotoxicity manifested as QT prolongation and arrhythmias [107,108,109,110,111]. The present studies reveal that specific inhibition of Rlip-mediated dox transport by anti-Rlip antibodies caused a greater effect on dox retention in breast cancer compared with the cardiomyocyte cells. Because of the similarity in the effects of AA and Rlip depletion, our results suggest the combination of AA and dox may not be more cardiotoxic than dox alone, though a three-drug combination of dox, AA and Rlip inhibitors could pose a greater risk. If future in vivo animal studies confirm our findings, clinical trials would be justified to determine the optimal intake of AA for improving the therapeutic index of dox. This approach may be valuable clinically in Her2-positive breast cancers that are resistant to trastuzumab, pertuzumab or lapatinib.

## 5. Conclusions

Her2-amplified or overexpressing breast cancer comprises roughly 20% of breast cancers [112]. Though trastuzumab, pertuzumab and lapatinib are effective treatments, 13% of ER + Her2 + and 22% of ER-Her2 + patients are expected to die within 5 years. Most patients with metastatic Her2-overexpressing cancer will have progressive disease on first-line anti-Her2 therapy within 1 year [113] and on second-line therapy in less than 6 months [114]. The mechanism of action appears to be through synergistic increases in 4-HNE, a pro-apoptotic metabolite specifically derived from oxidation of Ω-6 fatty acids. Our studies indicate that Her2-overexpressing breast cancer cells are very sensitive to AA and that combined treatment of these patients with doxorubicin with judicious increases in the Ω-6 fatty acid content of the diet during dox chemotherapy could improve treatment efficacy without exacerbating cardiotoxicity. AA supplementation or Rlip inhibitors combined with PIK3CA inhibitors recently approved for Her2-negative breast cancer treatment [115,116] could expand the utility of these drugs for Her2-amplified breast cancer as well.

## Figures and Tables

**Table 1:** AR activity, GSTA4 enzyme activity and GSH content in SK-BR-3 and AU565 cells. AR activity was significantly higher in SK-BR-3 cells, and 4-HNE conjugation activity was significantly higher in AU565 cells, as calculated by an unpaired two-tailed Student *t*-test. GSH content was similar in both cell lines.

Activity/Level	SK-BR-3	AU565	*p-*Value
Aldose Reductase ^1^	43.2 ± 10.2	27.7 ± 6.3	<0.001
GSTA4 ^1^	6.0 ± 0.8	22.9 ± 2.0	<0.0001
GSH ^2^	23.0 ± 0.6	20.7 ± 0.5	<0.05

^1^ nmol/mg protein, and ^2^ µmol/mg protein.

**Table 2:** Effect of AA on expression of antioxidant genes in SK-BR-3 and AU565 cells ^##^.

	SK-BR-3	AU565
**Genes for Antioxidant Enzymes**		
*CAT*	0.96 ± 0.01 n.s.	0.99 ± 0.03 n.s.
*SOD1*	1.42 ± 0.03 ***	1.18 ± 0.13 n.s.
*SOD2*	0.81 ± 0.02 ***	0.93 ± 0.04 n.s.
*GPX1*	2.77 ± 0.09 ***	1.28 ± 0.33 n.s
**Genes for Anti-Electrophile Enzymes**		
*GSTA4*	0.98 ± 0.07 n.s.	1.12 ± 0.12 n.s.
*AKR1C3*	3.84 ± 0.18 ***	2.29 ± 0.04 ***
*AKR7A2*	0.66 ± 0.01 *	1.07 ± 0.04 n.s.
**Genes for Glutathione Synthesis and Loss**		
*GCLC*	1.92 ± 0.02 ***	1.32 ± 0.06 *
*GCLM*	1.83 ± 0.03 ***	1.60 ± 0.01 ***
**Transcriptional Regulator of Antioxidant Genes**		
*NFE2L2*	1.09 ± 0.10 n.s.	0.97 ± 0.03 n.s.

^##^ Cells were treated with 100 μM AA for 24 h before qRT-PCR was performed as described in Materials and Methods. mRNA expression of each gene in AA-treated cells was compared to that of vehicle-treated control cells (* = *p* < 0.01; *** = *p* < 0.001, by Student’s *t*-test. n.s. indicates not significant).

## Data Availability

The data presented in this study are available in the article and Supplementary Material.

## Supplementary Materials

The following are available online at https://www.mdpi.com/article/10.3390/cancers13246377/s1, Figures S1–S3: Full Western blots showing target expression of proteins.
