# Supplementary material for: Anticancer Activity of Ω-6 Fatty Acids through Increased 4-HNE in Breast Cancer Cells
Source: Cancers (Basel). 2021 Dec 20;13(24):6377. doi: 10.3390/cancers13246377 (PMC8699056; doi:10.3390/cancers13246377)
Supplement: Supplementary file 1 [file cancers-13-06377-s001.zip › cancers-1481173-supplementary.pdf]

Supplementary Materials: Anticancer Activity of Ω-6 Fatty Acids through Increased 4-HNE in Breast Cancer Cells

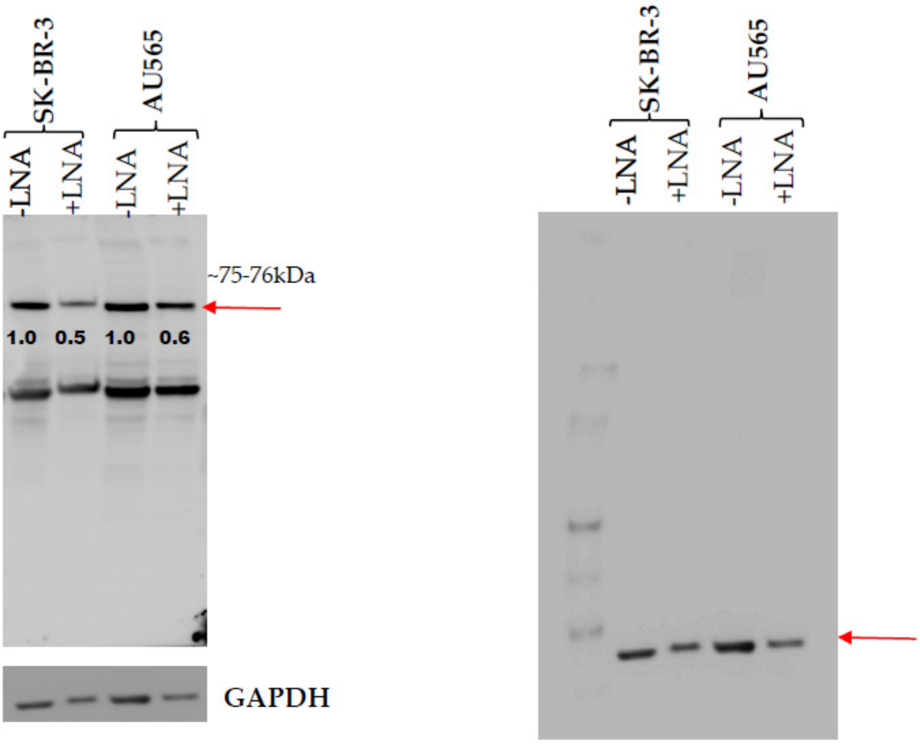

Figure S1. Supplemental data for Figure 2A. Efficiency of Rlip depletion was checked by western blot GAPDH was used as a loading control for total cellular proteins. Representative unprocessed western blots as shown in Figure 2A. Red Arrow show cropped area included in Figure 2A. Numbers below of each band represent protein band intensity ratios after normalization against GAPDH.

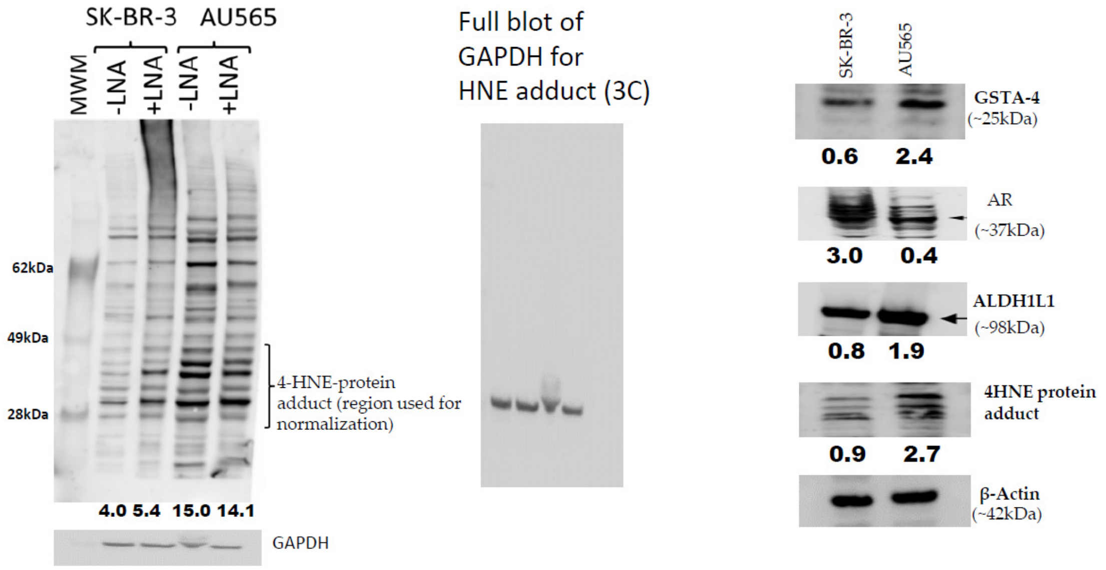

Figure S2. Supplemental data for Figure 3C,D. Numbers below of each blot represent protein band intensity ratios after normalization against β-Actin. Full blots of 3D are presented in next supplemental figures.

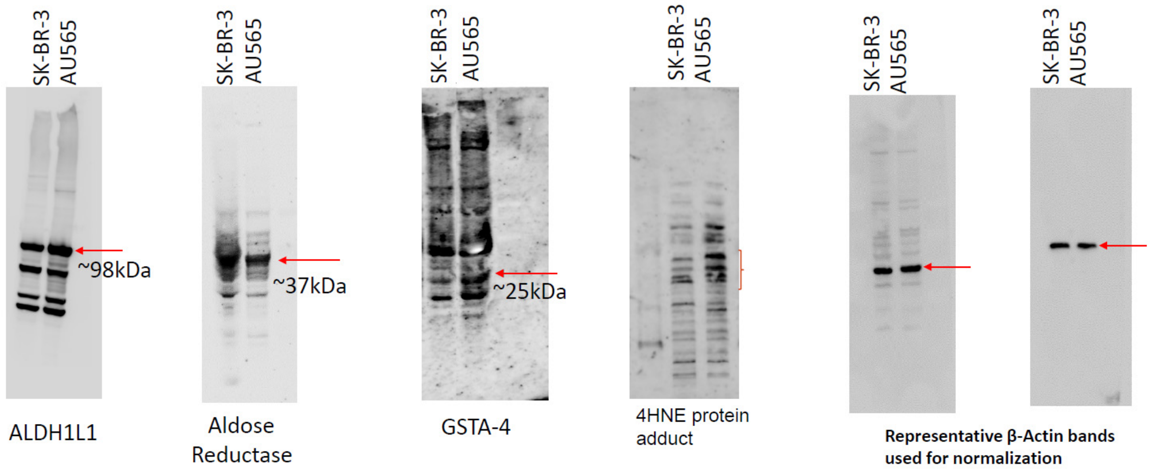

Figure S3. Supplemental data for Figure 3D.Representative unprocessed western blots of SK-BR-3 and AU565 cell lysate as shown in figure 3D. Red Arrow show cropped area included in Figure 3D.
